# Linking Oviposition Site Choice to Offspring Fitness in *Aedes aegypti*: Consequences for Targeted Larval Control of Dengue Vectors

**DOI:** 10.1371/journal.pntd.0001632

**Published:** 2012-05-01

**Authors:** Jacklyn Wong, Amy C. Morrison, Steven T. Stoddard, Helvio Astete, Yui Yin Chu, Imaan Baseer, Thomas W. Scott

**Affiliations:** 1 Department of Entomology, University of California Davis, Davis, California, United States of America; 2 United States Naval Medical Research Center Unit-6, Lima, Peru; 3 Fogarty International Center, National Institutes of Health, Bethesda, Maryland, United States of America; Mahidol University, Thailand

## Abstract

**Background:**

Current *Aedes aegypti* larval control methods are often insufficient for preventing dengue epidemics. To improve control efficiency and cost-effectiveness, some advocate eliminating or treating only highly productive containers. The population-level outcome of this strategy, however, will depend on details of *Ae. aegypti* oviposition behavior.

**Methodology/Principal Findings:**

We simultaneously monitored female oviposition and juvenile development in 80 experimental containers located across 20 houses in Iquitos, Peru, to test the hypothesis that *Ae. aegypti* oviposit preferentially in sites with the greatest potential for maximizing offspring fitness. Females consistently laid more eggs in large vs. small containers (β = 9.18, p<0.001), and in unmanaged vs. manually filled containers (β = 5.33, p<0.001). Using microsatellites to track the development of immature *Ae. aegypti*, we found a negative correlation between oviposition preference and pupation probability (β = −3.37, p<0.001). Body size of emerging adults was also negatively associated with the preferred oviposition site characteristics of large size (females: β = −0.19, p<0.001; males: β = −0.11, p = 0.002) and non-management (females: β = −0.17, p<0.001; males: β = −0.11, p<0.001). Inside a semi-field enclosure, we simulated a container elimination campaign targeting the most productive oviposition sites. Compared to the two post-intervention trials, egg batches were more clumped during the first pre-intervention trial (β = −0.17, P<0.001), but not the second (β = 0.01, p = 0.900). Overall, when preferred containers were unavailable, the probability that any given container received eggs increased (β = 1.36, p<0.001).

**Conclusions/Significance:**

*Ae. aegypti* oviposition site choice can contribute to population regulation by limiting the production and size of adults. Targeted larval control strategies may unintentionally lead to dispersion of eggs among suitable, but previously unoccupied or under-utilized containers. We recommend integrating targeted larval control measures with other strategies that leverage selective oviposition behavior, such as luring ovipositing females to gravid traps or egg sinks.

## Author Summary

Controlling the mosquito *Aedes aegypti*, the predominant dengue vector, requires understanding the ecological and behavioral factors that influence population abundance. Females of several mosquito species are able to identify high-quality egg-laying sites, resulting in enhanced offspring development and survival, and ultimately promoting population growth. Here, the authors investigated egg-laying decisions of *Ae. aegypti*. Paradoxically, they found that larval survival and development were poorest in the containers females most often selected for egg deposition. Thus, egg-laying decisions may contribute to crowding of larvae and play a role in regulating mosquito populations. The authors also tested whether removal of the containers producing the most adult mosquitoes, a World Health Organization-recommended dengue prevention strategy, changes the pattern of how females allocate their eggs. Elimination of the most productive containers led to a more even distribution of eggs in one trial, but not another. These results suggest that behavioral adjustments by egg-laying females may lessen the effectiveness of a common mosquito control tactic. The authors advocate incorporating control strategies that take advantage of the natural egg-laying preferences of this vector species, such as luring egg-laying females to traps or places where their eggs will accumulate, but not develop.

## Introduction

At present, dengue virus transmission can be controlled or prevented only through suppressing mosquito vector populations [1]. Even with the advent of a licensed dengue vaccine, which is anticipated by 2015 [2], vector control will remain a necessary component of any sustainable program to eliminate dengue transmission in endemic areas or prevent virus introduction into new areas [3]. Unfortunately, few contemporary dengue control programs have achieved the high thresholds of vector population suppression (estimated to be >90% at some locations [4], [5]) needed to prevent epidemics [6]. Controlling *Aedes aegypti*, the primary dengue vector worldwide, is challenging because it is well-adapted to the domestic environment [7], [8]. Adult mosquitoes rest indoors on clothing and underneath furniture, where they are difficult to reach using traditional aerosol or residual insecticides [7], [9]. Furthermore, females deposit their eggs in a wide assortment of man-made containers, ranging from water storage drums to discarded bottles and cans, making exhaustive larval control impractical in most cases [4], [10], [11].

*Ae. aegypti* productivity tends to be clustered at most field locations, with the majority of the adult population emerging from a small subset of water-holding containers [10]–[12]. Thus, targeting larviciding and container elimination efforts to these most productive containers may substantially improve the efficiency and cost-effectiveness of dengue control [13]. Proponents of targeted larval control predict that elimination of containers producing, for example, 80% of pupae will lead to a sustained linear reduction in the total adult density [10]. This expectation is based, however, upon two key assumptions: (1) all available *Ae. aegypti* larval development sites are already at carrying capacity and (2) oviposition behavior has little impact on population dynamics [10]. Field evaluations of targeted larval control programs have yielded mixed outcomes. Investigators in Myanmar and the Philippines reported nearly linear reductions (73–77%) in the *Ae. aegypti* Pupae per Person Index (PPI) after 5 months [12]. In Thailand, however, only a 15% reduction in PPI was observed after implementing a targeted control campaign designed to eliminate 80% of pupal production. In Iquitos, Peru, a 236% increase in PPI was noted after an intervention designed to eliminate 92% of pupal production [12]. Thus, the efficacy of targeted larval control varies substantially between settings and likely depends upon details of *Ae. aegypti* ecology and population dynamics at the local scale.

Selection of an oviposition site by a female mosquito directly affects offspring survival and growth [14]–[16], and has consequences for population dynamics [17]. Because evolutionary theory predicts that animals should act to maximize their reproductive success, egg-laying females are expected to select the most suitable sites for their offspring based on reliable cues of habitat quality [18]–[20]. Whether and how female *Ae. aegypti* select oviposition sites, the impact of oviposition decisions on offspring fitness, and how females adjust to changes in oviposition site availability will affect the validity of the two key assumptions underlying targeted larval control. Previously, we demonstrated that free-ranging *Ae. aegypti* in Iquitos actively select egg-laying sites [21]. In particular, females exhibited a preference for containers holding conspecific larvae and pupae. Container characteristics of secondary importance included large size, abundant organic material, and exposure to sunlight [21].

In the present study, we assessed whether *Ae. aegypti* oviposition site choice is correlated with offspring performance. We tested the prediction that females will lay more eggs in containers in which more juveniles successfully complete development and grow to large adult size, two important components of mosquito fitness [22]–[24]. We also investigated how individual females partition their egg batch among available containers. We predicted that, prior to targeted container elimination, individual females would cluster their egg batch in a preferred container, but switch to spreading their eggs widely among more remaining, available containers if preferred sites were eliminated. By examining whether *Ae. aegypti* females adjust their egg-laying strategies in response to environmental change as well as the implications of oviposition site choice for population dynamics, we hope to better understand why targeted larval control measures may not achieve the desired level of population reduction in some settings. Ultimately, we expect our detailed findings on *Ae. aegypti* behavior to provide insight for the development of improved strategies for vector population suppression.

## Materials and Methods

### Ethics statement

Households included in our field experiment were selected based on the home owners' willingness to participate. After explanation of study objectives and procedures, verbal consent was obtained from the head of each household. We did not collect information on household residents. Our study was approved by the local Ministry of Health, Dirección Regional de Salud-Loreto. Institutional Review Boards (IRBs) from the University of California, Davis and the United States Naval Medical Research Center (Project #: PJT-NMRCD.032) determined that our study did not meet the definition of human subjects research and IRB approval was, therefore, not required. A waiver of IRB approval was granted by the UC Davis IRB for feeding laboratory-reared mosquitoes on humans.

### Study location

Our study was conducted in Iquitos (73.2°W, 3.7°S, 120 m above sea level), a city of approximately 380,000 people in northeast Peru. Iquitos is located at the confluence of the Amazon, Nanay, and Itaya Rivers in the Department of Loreto and has been described in detail previously [25]–[27]. Daily air temperature, relative humidity, and rainfall data collected from a National Oceanic and Atmospheric Administration meteorological station located at the airport (∼6 km from the city center) demonstrated that the climate of Iquitos is relatively consistent year round, with rain falling during all months and small fluctuations occurring in temperature and relative humidity [28], [29]. Our experiments took place during August to November 2008. During these months, mean temperature (± SD) was 26.2±1.3°C, mean relative humidity (± SD) was 81.2±5.1%, and mean daily rainfall (± SD) was 6.0±12.0 mm [28].

### Establishing *Ae. aegypti* families

Both experiments conducted during this study (described below) required genotyping mosquitoes to match them to parents. We established 18 *Ae. aegypti* family lines in the field laboratory by collecting *Ae. aegypti* eggs (F_0_ generation) from 36 households across 18 neighborhoods in Iquitos. Because our goal was to make these families easily distinguishable, each family originated from a different neighborhood (males and females collected >100 m apart to avoid inbreeding) to maximize the number of alleles shared within a family and minimize alleles shared between families. Field-collected eggs were hatched by immersion in hay infusion overnight and larvae reared according to the standardized protocol described by Wong et al. [29]. Throughout the rearing process, mosquitoes were kept separated by collection house and date. Paired matings were set up as detailed by Wong et al. [30] and all F_0_ mosquitoes were assigned unique identifying numbers.

Females were offered an opportunity to imbibe blood from a human daily, but were not fed sugar (see [30]). F_1_ eggs were collected daily, labeled by the mother's identifying number, allowed to embryonate in a moist chamber for 48 hrs, dried for storage, and later hatched for experiments. Upon completion of three gonotrophic cycles or death, F_0_ parents were transferred to 1.5 mL plastic vials filled with 96% ethanol and stored at −20°C for subsequent genotyping.

### Experimental set-up

#### Preference–performance field experiment

**Figure 1:**
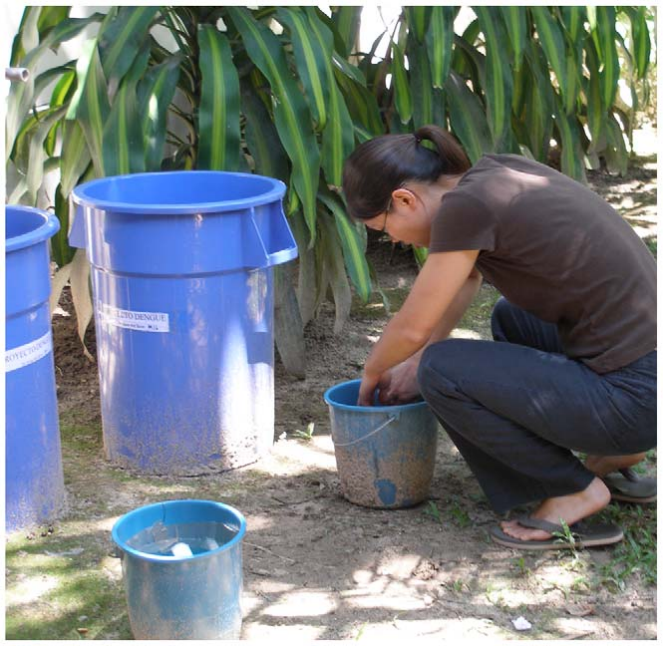
Experimental oviposition containers. Four blue containers made of rigid plastic were set out per house. Two containers were large trash cans (40 cm diameter×70 cm height) and two containers were small buckets (21 cm diameter×23 cm height). Container size was crossed with fill method (manually filled vs. unmanaged) to create four different treatments.

To assess *Ae. aegypti* oviposition preferences in the field, four blue containers made of rigid plastic were placed in the yards of 20 central Iquitos households and monitored daily for *Ae. aegypti* eggs over approximately one month. Ten houses were included in the study during August 2008 and another ten houses from mid-September to mid-October 2008. Container size (large trash can [40 cm diameter×70 cm height] vs. small bucket [21 cm diameter×23 cm height]) was crossed with fill method (manually filled vs. unmanaged) to create four different container treatments (Figure 1). We chose to manipulate these two variables because they were determined to be associated with oviposition choice in our previous study [21]. Effects of larval predators on oviposition site choice could not be examined because domestic containers in Iquitos generally lack predators such as copepods or fish (ACM and JW, unpublished data). Containers were arranged 0.5 m apart, filled to 66% capacity with tap water, and lined with strips of brown paper towel as a removable oviposition substrate. Manually filled containers were maintained with clean water every day by gently wiping the inside surface of containers by hand to dislodge bacteria and algae and then exchanging 75% of the water in the container for clean tap water. This was done to simulate daily water usage by Iquitos residents. Unmanaged containers were filled to 66% capacity with tap water on the first day, allowed to accumulate organic matter for the entire month and never cleaned. Paper strips from all containers were examined for *Ae. aegypti* eggs daily between 09:00 and 12:00h. If eggs were present, new paper liner was exchanged. Paper with eggs was transported to the field laboratory where eggs were counted under a dissecting microscope at 20× magnification.

**Figure 2:**
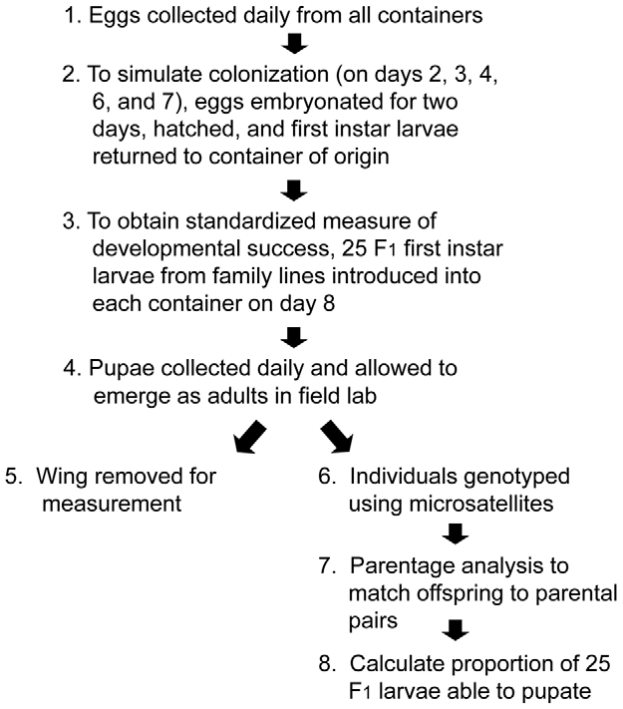
Flowchart of preference-performance field experimental design. Four container treatments were set out in each of 20 houses in Iquitos, Peru (80 containers total). Larvae originating from field-collected eggs (for the purpose of monitoring oviposition) were re-introduced into the same container to simulate colonization. Twenty-five F_1_ larvae from laboratory families were introduced into each container on day 8 to compare juvenile developmental success.

To assess the fitness of *Ae. aegypti* developing in these experimental containers, first instar larvae from two sources (from the same container and a laboratory family line) were introduced and their development monitored (Figure 2). To imitate natural container colonization by *Ae. aegypti*, eggs collected from containers were allowed to embryonate in a moist chamber for two days in the field laboratory and then were hatched by immersion in hay infusion overnight. On the following day, all first instar larvae were re-introduced into the same containers from where eggs originated three days prior. This procedure was repeated so that individuals collected as eggs on days 2, 3, 4, 6, and 7 were re-introduced into containers on days 5, 6, 7, 9, and 10, respectively. Due to this process, different numbers of larvae were introduced into each container depending on the number of eggs laid. To obtain a standardized measure of developmental success, we introduced 25 first instar F_1_ larvae from the above-described *Ae. aegypti* family lines into each container on day 8. After day 10, no additional larvae were added. Larval competition is asymmetric for *Ae. aegypti*, with strong effects expected on early instars, but no discernable effect upon later instars [31]. We stopped adding larvae back into experimental containers after day 10 because we assumed they would have minimal impact on the fitness of larvae introduced earlier.

All containers were monitored daily for *Ae. aegypti* pupae, which were transferred to Whirl-Pak bags (Nasco, Fort Atkinson, WI) and labeled by house, container, and date of collection. At the field laboratory, pupae were counted and placed in clear plastic vials (2.5 cm diameter×7 cm height) for adult emergence (up to 20 pupae per vial). Adults were mouth aspirated into 1 pint paper cartons and then killed by freezing at −20°C for 30 min. We assigned a unique code to each adult mosquito, determined its sex, and mounted one wing on a slide using double-sided tape. Wing length, used as a proxy for body size [32], [33], was measured using a DC5-420T digital microscope (National Optical, San Antonio, Texas) and Motic Images 2.0 software (Motic, Richmond, Canada). All measurements were made from the axillary incision to the wing tip, excluding fringe scales [34]. The remainder of the body was stored in 96% ethanol in a 1.5 mL plastic vial at −20°C and later genotyped to identify individuals from the established family lines (described below). Pupae that died before emergence were also assigned a code, stored in ethanol, and genotyped. Containers were monitored for eggs and pupae for 23 days during August and for 28 days during September and October. At the conclusion of the study, all larvae remaining in containers were enumerated to instar based on size and morphology [35].

#### Experimental targeted container elimination

Female *Ae. aegypti* were released into a semi-field enclosure to observe how individuals distribute their eggs before vs. after targeted container elimination. An enclosure (13.5 m×4.7 m×2.7 m height) was built inside a vacant house and yard in central Iquitos. Because the house shared brick walls with neighboring houses on both sides (typical for Iquitos), a wooden scaffold was erected against the inside walls and fine nylon mesh was used to cover the scaffold. The enclosure was extended out into the yard. The windows between the house and yard were left open to allow free movement of mosquitoes indoors and outdoors within the enclosure.

**Figure 3:**
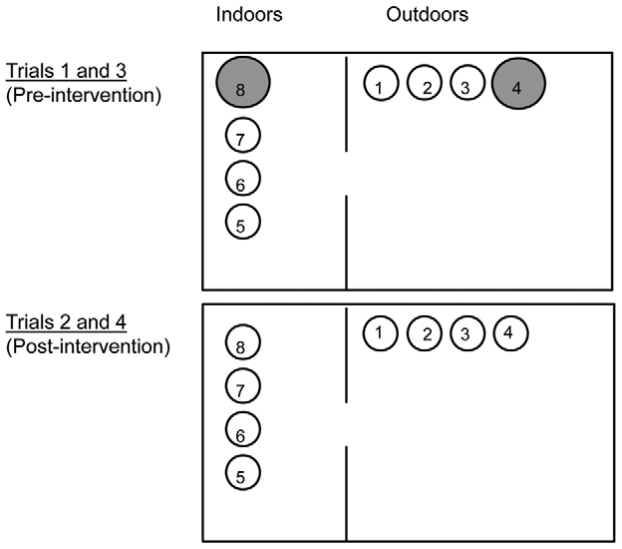
Diagram of oviposition sites within semi-field enclosure. Trials 1 and 3 were pre-intervention trials during which females were presented with two large unmanaged containers (grey circles) and six small manually filled containers (white circles). Trials 2 and 4 were post-intervention trials during which females had access to eight small manually filled containers. Containers 1–4 were located outside in the yard and containers 5–8 were inside the house. The windows were left open to allow free movement of mosquitoes indoors and outdoors. Containers are not drawn to scale with the house.

Eight of the blue plastic water-filled containers from the previous field experiment were lined with strips of brown paper towel and placed in the enclosure to serve as potential oviposition sites (Figure 3). Four containers were grouped (0.5 m apart) inside the house and four grouped (0.5 m apart) outside in the yard. To simulate natural oviposition patterns, we used a combination of the most preferred (large, unmanaged) and least preferred (small, manually filled) containers. Under the pre-intervention scenario (trials 1 and 3), one of the four containers in each group was of the most preferred type (large and filled with water that had accumulated organic debris for two weeks) and the remaining containers were of the least preferred type (small and filled with fresh tap water on the first day). Under the post-intervention scenario, (trials 2 and 4), all eight oviposition containers were of the least preferred type. In the post-intervention scenario, we chose to replace the two most preferred containers with two least preferred containers so that the total number of containers remained consistent between trials. Thus, any alterations in oviposition patterns would be attributable to changes in the characteristics of available containers, rather than a difference in the total number of containers. Also, we chose to conduct separate trials for the pre- vs. post-intervention scenarios rather than simply switching half way through each trial. Because insect selectivity for oviposition sites may change with age [36], switching experimental treatments after a set amount of time could have confounded results due to container changes with those due to female aging.

To rear females for release into the enclosure, F_1_ eggs were hatched and larvae reared as described above. Within two hours of emergence, adult females had one rear leg removed as described by Wong et al. [30]. Legs were placed in individual 1.5 mL plastic vials filled with 96% ethanol and stored at −20°C for subsequent genetic analyses. Females were then aspirated into individual 1 pint paper cartons with a single unmated male sibling. Removal of the leg did not noticeably affect longevity, mating, fecundity, or oviposition behavior of female *Ae. aegypti* in our previous laboratory study [30]. Females were offered human blood once per day, as previously described, on the second and third days after emergence. On the fourth day after emergence, 10–13 gravid females (each from a different family) were released into the semi-field enclosure. Males from those mated pairs were killed by freezing, preserved in individual 1.5 mL plastic vials filled with 96% ethanol, and stored at −20°C until used for genetic analyses.

During each of four trials, gravid females were released in the center of the enclosure at 12:00h. Females were given the opportunity to blood feed daily on one of the investigators (J.W.) for 30 minutes between 10:00 and 12:00h. During this time, the eight containers were checked for *Ae. aegypti* eggs daily. To compensate for high female mortality during trial 2 (few eggs collected on the first two days), we introduced into the enclosure eight additional gravid females (from the same cohort of F_1_ mosquitoes) on the third day after the initial release. Because these supplemental mosquitoes had spent three extra days in the laboratory, they were released during their second gonotrophic cycle.

If eggs were present in containers, the paper liner was changed. Papers with eggs were sealed in plastic bags and labeled with the container number and date. At the field laboratory, eggs were counted under a dissecting microscope at 20× magnification, were allowed to embryonate for 48 hrs, and then hatched by immersion in hay infusion overnight. Because *Ae. aegypti* eggs hatch in installments [35], any unhatched eggs were dried and the hatching process repeated twice for a total of three inundations. Larvae were kept separated by collection date and container, and reared to the pupal stage according to our standardized protocol [29]. All pupae and any larvae that died were preserved in 96% ethanol in 1.5 mL plastic vials and kept at −20°C until used for genetic analyses.

Trials 1, 2, and 3 each lasted ten days. Trial 4 ended after seven days due to high female mortality. We suspect that insecticide spraying in one of the neighboring houses reduced survival of adult *Ae. aegypti* during our last trial. At the end of each trial, we attempted to collect any remaining females by landing catch. Sequential trials were separated by gaps of 9–11 days without human presence in the enclosure to minimize survival of adult females between releases.

### Meteorology

Data loggers were used to record weather variables once per hour. During the field experiment, Hobo ProV2 data loggers (U23-001) were deployed in 14 of the 20 houses (attached to the side of a container) to monitor ambient temperature and relative humidity (Onset Computer Corporation, Pocasset, MA). In the same houses, Hobo Pendant loggers (UA-002-64) were placed inside containers to monitor water temperature. We did not have enough data loggers to monitor weather at all 20 houses, but based on previous experience we expected that temperatures would be consistent across the city. Within the semi-field enclosure, loggers were used to record air temperature, relative humidity, and water temperature indoors and outdoors once per hour.

### Genotyping and parentage analysis

All specimens from this study were transported to the University of California, Davis (UCD) for DNA extraction and genetic analysis. DNA from adults used in paired laboratory matings (to establish families) was purified by potassium acetate/ethanol precipitation [37]. DNA from legs of released females was isolated using the same method, with the exception that reagents were used at 50% volume. Due to the large number of experimentally collected mosquitoes (from the field or semi-field enclosure), DNA from these individuals was purified using the automated BioSprint 96 DNA extractor and reagents from the BioSprint 96 Kit (Qiagen, Valencia, CA).

Individuals were genotyped at ten microsatellite loci using fluorescent-labeled forward primers as described in Wong et al. [30]. Polymerase chain reaction (PCR) products were diluted 1∶60 in ddH_2_O and submitted to the College of Agriculture and Environmental Sciences Genomics Facility at UCD (http://cgf.ucdavis.edu/home/) for fragment analysis on an ABI 3730 XL capillary sequencer (Life Technology Corp., Carlsbad, CA). Resulting chromatograms were analyzed using ABI Peak Scanner™ software (Applera Corp., Norwalk, CT). Exclusion-based parentage analysis was performed using PROBMAX version 1.2 [38] to identify offspring of parental pairs [30].

### Data analysis

#### Female oviposition preference

All regression analyses were conducted using R version 2.8.1 [39]. To identify patterns in female oviposition preference among the four container treatments, a linear mixed effects model was fit to the data using the “nlme” package [40]. The response variable, eggs per container, was summed over each week and normalized by container circumference. These data were square root transformed so the resulting model conformed to normality assumptions. Trial, week, larval density, container size, fill method, and size by fill interaction were included as fixed effects. House was included as a random effect to account for repeated sampling. Throughout our analyses, larval density was re-expressed as a percentage of the mean density among containers of the same size and fill method. This was done to avoid colinearity between larval density and container size and/or fill method, and to examine the impact of variation in larval density within each treatment. Model selection was carried out using the likelihood ratio test and the final model was fit using restricted maximum likelihood estimation [41].

#### Performance of immature *Ae. aegypti*

For each container, the proportion of F_1_ mosquitoes from lab families (out of 25 individuals) able to pupate within the observation period was recorded as the response variable (standardized pupation probability). F_1_ individuals that died and those that remained alive as larvae at the conclusion of the experiment were considered as failing to pupate. These data were analyzed by fitting a binomial generalized linear model using container size, fill method, size by fill interaction, larval density, and house as predictor variables. To adjust for underdispersion, standard errors were corrected using a quasi- generalized linear model with dispersion factor = 0.18. Tukey's multiple comparison tests were applied using the “multcomp” package [42] to identify differences in container treatment effects.

Because standardized pupation probability was small (or zero) for some containers, we analyzed wing length data for all mosquitoes collected during the study, rather than only those originating from laboratory families. Wing lengths were analyzed separately for male and female mosquitoes using linear mixed effects models. Fixed effects included trial, larval density, container size, fill method, and size by fill interaction. House was used as a random effect. Model selection was carried out using the likelihood ratio test and the final model was fit using restricted maximum likelihood estimation.

#### Experimental targeted container elimination

Because 1,072 eggs collected from within the semi-field enclosure (26.2%) failed to hatch, we were unable to genotype and assign parentage to unhatched eggs. For that reason, analysis of egg distribution data was carried out in two steps: (1) testing for differences in egg dispersion patterns by individual females (restricted to genotyped offspring only) and (2) testing for differences in the probability that containers received eggs (included all eggs, genotyped or not).

To quantify the evenness with which individual *Ae. aegypti* dispersed their eggs among the eight containers, we calculated the Shannon equitability index [43] for each female over each gonotrophic cycle:
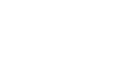
In this equation, *p_i_* represents the proportion of a female's offspring deposited in container *i*, and *s* denotes the total number of containers (*s* = 8). This index ranges from 0 to 1 and takes into account both the number of eggs laid and their relative distribution among containers. *J* reaches a maximum when offspring are evenly distributed among all containers and a minimum when all offspring are concentrated within a single container. Because containers receiving zero eggs were problematic for analysis, all egg counts were transformed by adding 0.01.

Using the Shannon equitability index as the response variable, a binomial generalized linear model was fit to our data to identify the factors influencing how evenly females distribute their offspring. Trial, gonotrophic cycle number, and female were included as potential covariates. To adjust for underdispersion, standard errors were corrected using a quasi- generalized linear model with dispersion factor = 0.13.

To identify the factors affecting whether or not a container received eggs, a logistic regression model was fit to the egg data (1 = eggs deposited in container that day, 0 = no eggs deposited in container that day). Days on which no females oviposited (no oviposition site choices were made) were excluded from analysis. Enclosure treatment (pre- vs. post-intervention), container location (indoors vs. outdoors), container type (large unmanaged vs. small manually filled), and day were included as fixed effects. Trial was examined as a potential random effect. For all analyses, final models were validated by plotting the normalized residuals against fitted values and all covariates to ensure that no patterns were evident.

## Results

### Meteorology

During the field study, mean air temperature, water temperature, and relative humidity were consistent across houses and between the two trial periods (Table S1). Mean air temperature ranged from 26.6±1.9°C to 28.1±2.7°C. Water temperature was similar to air temperature, but exhibited less fluctuation throughout the day. Mean relative humidity ranged from 76.3±6.1% to 82.8±6.1%.

Within the semi-field enclosure, air temperature, water temperature, and relative humidity were also similar between trials (Table S2). Mean air temperature ranged from 27.3±0.8°C to 29.3±1.0°C indoors and from 26.4±1.2°C to 29.7±1.3°C outdoors. In general, temperatures fluctuated less in water compared to air, and less indoors compared to outdoors. Mean relative humidity ranged from 70.8±4.8% to 83.6±4.9%.

### Female oviposition preference

**Figure 4:**
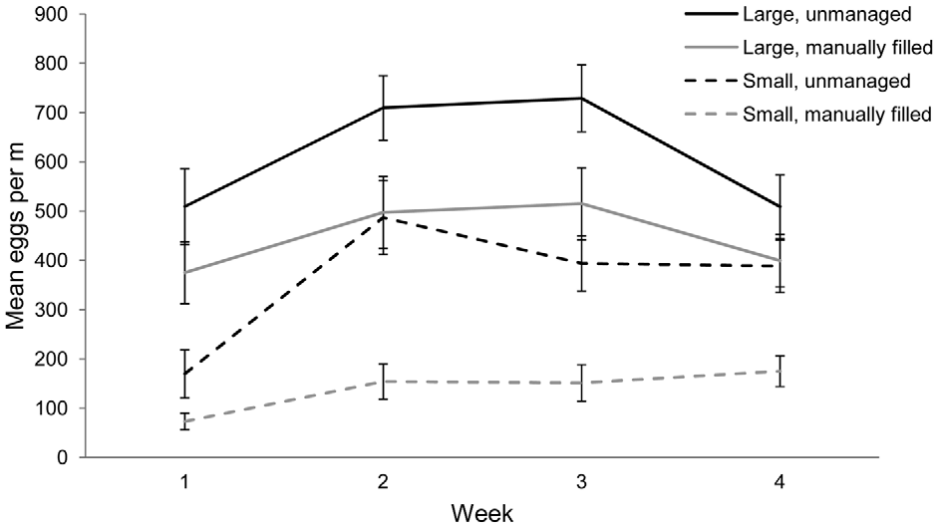
Oviposition by container type. Mean (± SE) number of eggs laid by *Ae. aegypti* in four container treatments in Iquitos, Peru (80 containers located in 20 houses). Daily egg counts for each container were summed over each week and divided by container circumference.

Data on the mean number of eggs deposited per container per week are shown in Figure 4. The optimal model included a random effect due to house and fixed effects due to container size, fill method, larval density, and week (Table 1). In general, more eggs were laid in large vs. small containers, in unmanaged vs. manually filled containers, with increasing larval density, and to a lesser extent, with week. There was no significant effect of trial (likelihood ratio = 0.57, p = 0.45) or container size by fill interaction (likelihood ratio = 1.29, p = 0.256).

**Table 1:** Parameter coefficients for model predicting number of eggs laid per container per week.

Random effects	Standard deviation
House	3.79
Residual	5.14

Fixed effects	Coefficient	Standard error	t value	Pr>t
Size (large)	9.18	0.68	13.60	<0.001
Fill method (unmanaged)	5.33	0.67	8.01	<0.001
Larval density	5.54	1.11	4.98	<0.001
Week	1.36	0.31	4.38	<0.001

A linear mixed effects model was fit to the data using house as a random effect (n = 80 containers).

### Pupation by genetically identified F_1_ *Ae. aegypti*

**Figure 5:**
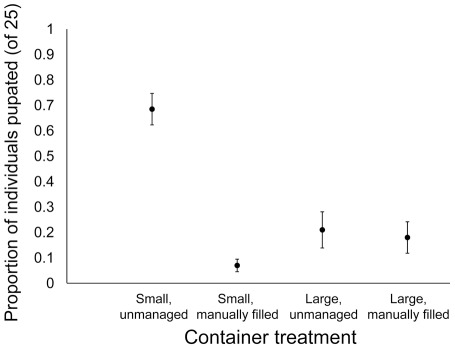
Standardized pupation probability by container type. Mean (± SE) proportion of larvae from laboratory families that pupated during the experiment. Proportions were calculated by genotyping all collected pupae to identify those originating from laboratory families (25 F_1_ first instars introduced into each container). Data came from eight houses during the first trial.

A total of 3,263 pupae were collected from all containers located in the 20 households (mean [± SE] = 40.7±7.3 pupae per container; range = 0 to 384). More pupae were collected from large unmanaged containers (n = 1,933 pupae) than any other container treatment (Figure S1). Due to the time-intensive nature of genotyping all mosquitoes in order to identify those introduced from established families (25 F_1_ larvae introduced per container), standardized pupation probability was calculated for *Ae. aegypti* from containers in eight houses from the first trial (Figure 5). In these eight households, mean (± SE) larval density just prior to F_1_ introduction was 2.56 (±0.83) larvae per L in large unmanaged containers, 0.76 (±0.37) larvae per L in large manually filled containers, 0.75 (±0.32) larvae per L in small unmanaged containers, and 0 larvae per L in small manually filled containers. Of the 996 pupae genotyped, we matched 231 individuals to parental pairs from established families (mean [± SE] = 7.2±1.3 matched individuals per container; range = 0 to 23).

Pupation probability was significantly influenced by treatment (container size by fill method interaction) and house. First instar larvae introduced into small unmanaged containers exhibited significantly higher probability of pupation (β = 3.37, p<0.001) compared to individuals in the three other container types. No differences in pupation probability were observed among individuals developing in small manually filled containers compared to large unmanaged (β = −2.37, p = 0.214) or large manually filled containers (β = −1.79, p = 0.479). There was also no difference in pupation probability between individuals from large containers, regardless of fill method (β = 0.58, p = 0.811). Within each container treatment, we found no significant effect of larval density on pupation rates (likelihood ratio = 0.67, p = 0.414).

### Size of adult *Ae. aegypti*

**Figure 6:**
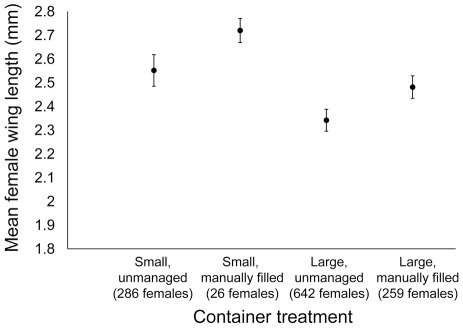
Adult size by container type. Mean (± SE) wing length of females developing in four container treatments.

Mean wing length of female mosquitoes collected from all 20 houses are shown in Figure 6. Wing lengths of males followed a similar pattern (Figure S2). Female wing lengths ranged from 1.85 to 3.23 mm (median = 2.51 mm) and wing lengths of males ranged from 1.55 to 2.56 mm (median = 2.00 mm). The optimal mixed effects models included a random effect due to house and fixed effects due to container size, fill method, and larval density (Table 2). Female wing length decreased significantly among *Ae. aegypti* developing in large vs. small containers, in unmanaged vs. manually filled containers, and with increasing larval density. Similar patterns were observed for males, with wing length decreasing in large containers, in unmanaged containers, and with increasing larval density. For both sexes, there was no significant effect of trial (females: likelihood ratio = 1.72, p = 0.190; males: likelihood ratio = 1.38, p = 0.24) or container size by fill interaction (females: likelihood ratio = 0.25, p = 0.803; males: likelihood ratio = 0.61, p = 0.435).

**Table 2:** Parameter coefficients for models predicting adult wing lengths.

Females	
Random effects	Standard deviation
House	0.20
Residual	0.14

Fixed effects	Coefficient	Standard error	t value	Pr>t
Size (large)	−0.19	0.05	3.80	<0.001
Fill method (unmanaged)	−0.17	0.01	−15.98	<0.001
Larval density	−0.16	0.01	−13.54	<0.001

Males	
Random effects	Standard deviation
House	0.11
Residual	0.10

Fixed effects	Coefficient	Standard error	t value	Pr>t
Size (large)	−0.11	0.03	3.15	0.002
Fill method (unmanaged)	−0.11	0.01	−13.92	<0.001
Larval density	−0.08	0.01	−9.20	<0.001

Linear mixed effects models were fit separately for females and males using house as a random effect (n = 80 containers).

### Experimental targeted container elimination

The numbers of females released, eggs collected, and offspring genotyped during each trial within the semi-field enclosure are shown in Table 3. The total number of eggs collected decreased steadily during each successive trial. Detailed results regarding on which days and in which containers individual females laid their eggs (those that could be genotyped) are displayed in Figure S3.

**Table 3:** Set up and results for targeted container elimination trials inside enclosure.

				Total		Total	Mean no. egg	Mean no.
	Enclosure	Females	Females	eggs	No. offspring	egg	batches per	eggs per
Trial	treatment	released	laid egg*	collected	genotyped (%)	batches*	female* (± SD)	batch* (± SD)
1	Pre-intervention^a^	12	8	1,631	1,231 (75.5%)	20	2.5 (±1.2)	61.6 (±21.8)
2	Post-intervention^b^	12 (8)#	4 (7)#	1,058	810 (76.6%)	18	1.6 (±0.7)	44.9 (±23.7)
3	Pre-intervention^a^	13	6	770	620 (80.5%)	12	2.0 (±1.1)	51.5 (±24.5)
4	Post-intervention^b^	13	9	628	354 (56.4%)	11	1.2 (±0.4)	32.2 (±25.8)

Offspring were genotyped using microsatellite markers and matched to parental pairs in order to track when and where eggs were laid by individual females.

a Two large unmanaged containers and six small manually filled containers available within enclosure.

b Eight small manually filled containers available within enclosure.

*: Estimates based on genotyped offspring (not including eggs that failed to hatch).

# Additional females (in parentheses) introduced into enclosure three days after initial release to compensate for high female mortality.

**Figure 7:**
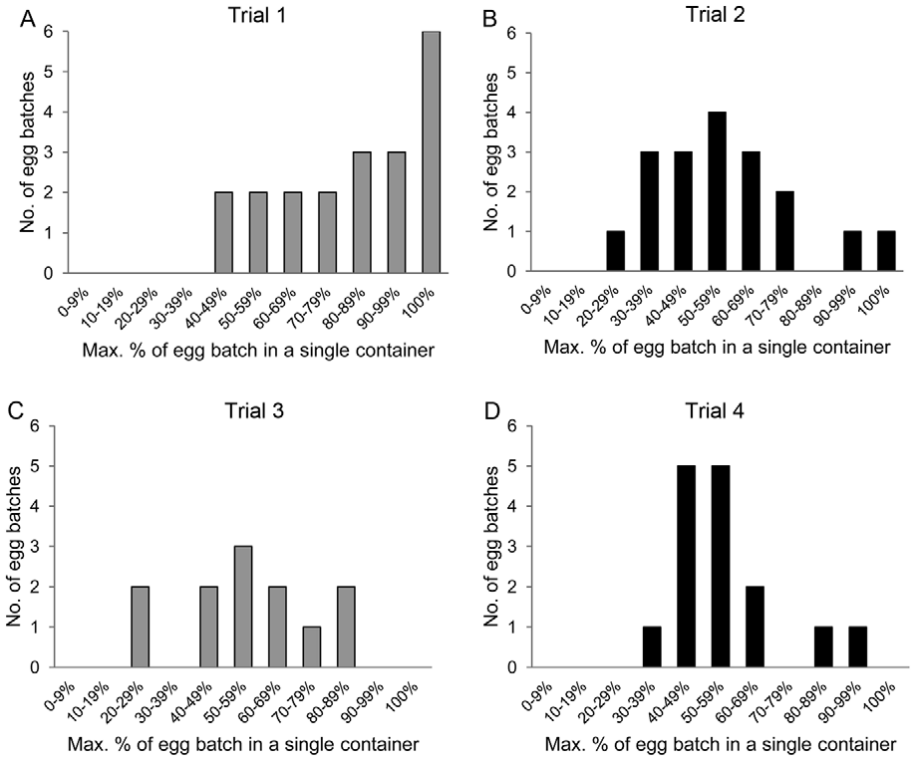
Concentration of eggs within a single container. Within the enclosure, the frequency distribution for the maximum proportion of each egg batch concentrated in any single container is shown for: A) trial 1 (pre-intervention), B) trial 2 (post-intervention), C) trial 3 (pre-intervention), and D) trial 4 (post-intervention). Preferred (large unmanaged) containers were available during pre-intervention trials (denoted by grey bars) but not during post-intervention trials (denoted by black bars).

Based on genotyped offspring, we calculated the largest proportion of each egg batch that was concentrated within a single container (Figure 7). During the first trial (pre-intervention), six egg batches were each aggregated within a single container (always in a large unmanaged container). Among all subsequent trials (pre- and post-intervention), there was only a single batch in which all eggs were deposited within a single container (trial 2, concentrated in a small manually filled container). In general, egg distribution was more clumped during the first trial compared to the later three trials.

**Figure 8:**
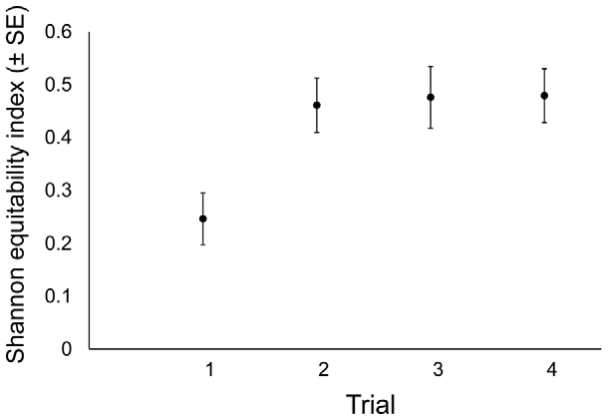
Egg dispersion by females inside enclosure. Mean values (± SE) of the Shannon equitability index for each semi-field trial. This index takes into account both the number of eggs laid and their relative distribution among containers. This index reaches the maximum value (1) when eggs are evenly distributed among all containers and the minimum value (0) when eggs are concentrated within a single container.

Values for the Shannon equitability indices for each trial are shown in Figure 8. In our model, Shannon indices were affected by trial, but not by gonotrophic cycle number or female (data not shown). Shannon equitability indices were significantly different between the two pre-intervention trials (trial 1 vs. trial 3, β = 0.18, p = 0.014), but not between the two post-intervention trials (trial 2 vs. trial 4, β = 0.02, p = 0.991). Individual females' egg batches were more clumped during trial 1 (pre-intervention) compared to the two post-interventions trials (β = −0.17, p<0.001). There was no difference, however, in Shannon equitability indices for trial 3 (also pre-intervention) compared to the two post-intervention trials (β = 0.01, p = 0.900).

When containers were examined daily for whether or not they received eggs (all eggs included, genotyped or not), the random effect of trial was not significant (intercept variance = 0). The probability that a container received eggs increased when containers were located indoors (β = 1.36, p<0.001) and if containers were large and unmanaged (β = 1.16, p = 0.012). The overall probability that any container received eggs increased during the post-intervention scenario (only small manually filled containers present in enclosure: β = 1.36, p<0.001).

## Discussion

When presented with a choice of four container types varying in size and organic content, wild female *Ae. aegypti* consistently deposited more eggs in large containers with abundant organic material. This behavior is expected to be adaptive, with females choosing sites based on cues of habitat quality. After monitoring the development of juvenile *Ae. aegypti*, however, we did not find a positive association between female egg-laying choice and juvenile growth or survival. The container type most preferred by ovipositing females (large unmanaged) produced individuals with low pupation probability and small adult body size. Pupation probability was highest among *Ae. aegypti* in small unmanaged containers, which received ample food and relatively few eggs, creating an environment consistent with low competitive pressure for food. In large unmanaged containers, we suspect that high food content was offset by high larval density. Large unmanaged containers may have quickly reached carrying capacity, so that F_1_ pupation rates were no better than in sites receiving little total food (manually filled containers). Prior to F_1_ introduction, mean larval density was 3.4 times greater in large unmanaged containers (2.56 larvae per L) compared to small unmanaged containers (0.75 larvae per L). To avoid colinearity with container size and fill method, we did not directly assess larval density as a predictor in our models. We instead examined relative larval density within each container treatment, but found no significant effect of larval density on pupation rates. The negative impact of high larval density was evident, however, in our analysis of *Ae. aegypti* body size. Large unmanaged containers yielded the smallest adult mosquitoes. Furthermore, within each of the four container treatments, body size clearly decreased with increasing larval density. Our result is consistent with previous field studies in Iquitos [26], Puerto Rico [44], and Thailand [5] that demonstrated negative relationships between the density of larvae in aquatic habitats and the size of emerging adults. Wing lengths of females collected during our study (range = 1.85 to 3.23 mm, median = 2.51 mm) were comparable to those reported by Schneider et al. [26] in Iquitos (range = 1.67 to 3.83 mm, median = 2.60 mm).

Mismatches between female oviposition preference and offspring performance have been reported for several insect species (e.g., [45], [46]), including mosquitoes [47]. Sub-optimal oviposition site selection may result from females' inability to predict stochastic events, sense determinants of site quality, or obtain complete knowledge of the environment [47]. Alternatively, apparent mismatches are sometimes attributed to experimental design and/or failure to examine important variables [46]. We attempted to simulate *Ae. aegypti* container colonization and water-use patterns typical of Iquitos, but our study was limited in some respects. During the re-introduction of larvae into containers to imitate colonization, eggs were hatched synchronously rather than gradually in installments, as is typical for *Ae. aegypti* [35]. The faster rate of larval introduction may have disproportionately increased levels of density-dependent competition in the most preferred containers (large unmanaged).

Containers occurring naturally in the field are likely to experience different rates of water evaporation and filling. This may result in dramatic fluctuations in larval densities, as well as variable cycles of desiccation and/or overflowing. To make our study design and analysis tractable, we artificially maintained stable water levels in our experimental containers. For species whose larvae develop in small containers and must mature before the habitat desiccates, maternal ability to assess water permanence would be favored [48]. It is possible that female *Ae. aegypti* evolved to detect cues associated with water permanence, and thus acted to trade off risks between desiccation and food competition for their progeny. Due to our experimental design, we were unable to assess the importance of container desiccation as a selective force in oviposition site choice. Such an investigation would require a detailed study on water dynamics of naturally-occurring (i.e., non-experimental) containers.

Previously, we observed that the majority of *Ae. aegypti* eggs tend to be aggregated within a small subset of containers. In addition, females were most likely to oviposit in sites that contained, or had recently contained, conspecific larvae and/or pupae [21]. These findings are consistent with other studies demonstrating that semiochemicals produced by conspecifics [49], [50] and conspecific-associated bacteria [51] act as oviposition attractants for *Ae. aegypti* (reviewed in [52]). During the present study, we did not attempt to isolate or identify these chemical mediators. Instead, our intention was to complement chemical ecologists' studies by investigating the consequences of conspecific attraction for *Ae. aegypti* offspring fitness and population dynamics. In our study, large aggregations of larvae in preferred containers led to the production of numerous small adults. For mosquitoes, adult body size can have important impacts on the rate of pupation growth and patterns of virus transmission. Small body size has been correlated with reduced life span and decreased fecundity for females and decreased mating success for males (e.g., [24], [53]–[56]). Female body size also exhibits a complex relationship with several components of vectorial capacity. A population dominated by small females, which are less susceptible to oral dengue infection [57] and less persistent in seeking blood meals [58], may serve to attenuate dengue transmission. On the other hand, small females must feed more frequently [59], [60], which could lead to increased rates of human-vector contact and enhance virus transmission.

Our results indicate that *Ae. aegypti* oviposition site choices that lead to crowding of larvae may play a role in population regulation by limiting the production and size of adults. In this situation, removal of the most productive containers would reduce adult abundance in the short term, but the long term population-level outcome would depend on the availability of alternative suitable oviposition sites in the area. If all water-filled containers are infested to their carrying capacity, targeted larval control is expected to result in a sustained, linear reduction in adult mosquito density [10]. On the other hand, if suitable unoccupied or under-utilized containers are available, targeted larval control could merely shift production to new containers over the next few generations. Results from our companion study indicated that, in Iquitos, containers suitable for *Ae. aegypti* development are frequently unoccupied (STS, unpublished). We predict that colonization of previously unoccupied sites could release large numbers of larvae from density-dependent food competition, eventually attenuating or undermining the immediate gains of targeted larval control. Results from a Brazilian field study support this idea. Maciel-de-Freitas and Lourenço-de-Oliveira [61] documented that elimination of the most productive container type (water tanks accounting for 72% of pupae) led to increased productivity from almost all other container classes, most notably in metal drums, which shifted from producing 3.5% to 30.7% of all pupae. Accompanied by this shift in productivity was a rebound in the adult densities to pre-intervention levels within 4–5 weeks. Only after eliminating both water tanks and metal drums (which were considered unimportant prior to the intervention) did investigators observe a long term drop in adult densities. The authors speculate that sustained reductions in *Ae. aegypti* densities were possible because of the similarity between water tanks and metal drums; both are large, typically shaded, perennial water storage containers. Even then, interventions that were designed to eliminate 75.9% of pupal production resulted in a 45.7% reduction in adult densities [61]. We suspect that in Iquitos and other locations where rain falls year round, large numbers of alternative containers and plasticity in *Ae. aegypti* oviposition behavior will render the long term results of targeted larval control less effective than anticipated.

The degree and speed of population recovery will also depend on whether females' egg distribution strategies are influenced by the characteristics of available containers. Inside the semi-field enclosure, egg distribution patterns were more aggregated for females during the first pre-intervention trial (trial 1), but not the second (trial 3), compared to the two post-interventions trials (trials 2 and 4). During trial 1, females frequently shared preferred oviposition containers, clustering the overall majority of eggs in these two sites. When only least preferred containers were available (post-intervention, trials 2 and 4), females were less likely to concentrate a large portion of their egg batch in any particular site, leading to a more even overall dispersion of eggs among containers. This pattern of spreading eggs evenly among sites, however, was also observed during our second pre-intervention trial (trial 3). Our mixed results suggest that egg distribution strategies are somewhat plastic and context-dependent. Differences between trials 1 and 3 may be the result of behavioral variation among individuals. Even individuals within a population are expected to vary in oviposition site selection strategies [62]. It is thus conceivable that individuals faced with similar environments could vary in their egg distribution strategies as well. Nonetheless, when we examined all eggs laid within the enclosure (genotyped or not), the overall probability that a container received eggs did increase during the post-intervention trials. The possibility that females may spread eggs more widely after elimination of the most productive containers is consistent with evidence from the field [61] and deserves further investigation.

A major shortcoming of this experiment was our inability to genotype offspring from eggs that failed to hatch. Overall, we were able to assign parentage to 74% of all offspring from the semi-field enclosure. This provides an informative, albeit incomplete, picture of oviposition patterns among the released females. If all unhatched eggs could be attributed to a few uninseminated females, we would expect our conclusions to be unbiased. Alternatively, if a proportion of every female's egg batch failed to hatch, this could lead us to underestimate the number of containers used by ovipositing females. We suspect that the true explanation lies somewhere between these two extremes. Another limitation of our study was that we substituted the two large containers with two small containers under the post-intervention scenario, which is unrealistic for a dengue control campaign. We took this step to prevent confounding between the effects of targeting specific containers as opposed to reducing container abundance in general. Targeted larval control campaigns are specifically directed at the small subset of most productive containers, so we would not expect overall container abundance to change dramatically. For this reason, we were more interested in how females responded to the non-availability of large containers rather than a reduction in container numbers. Had we been able to conduct more trials inside the enclosure, we would have examined effects of container removal without substitution, as well as effects of varying *Ae. aegypti* female density in the household.

Finally, all females in this experiment were confined to the one household within the semi-field enclosure. This design precluded us from testing whether female oviposition choices would be different if they had access to multiple houses and different container types, as occurs naturally in the field. We had originally planned to address that question during a field validation in which we would release females into the field and search for their progeny in the release house as well as neighboring houses. Due to a dengue-4 epidemic in Iquitos during fall 2008 [63], [64], however, we were unable to release mosquitoes to conduct this field validation.

We do not dispute that larval *Ae. aegypti* control should be practiced or that interventions such as container elimination, larviciding, and biological control are more cost effective when targeted to the most productive containers [12]. We suggest, however, that targeted larval control alone should not be relied upon as the predominant strategy to prevent dengue transmission. Due to the complexity of *Ae. aegypti* ecology and the low population threshold densities required for dengue transmission [4], [5], a combination of multiple control measures (e.g., container elimination, egg sinks, autodissemination of insect growth regulators, lethal ovitraps, etc.) will likely be necessary to produce an epidemiologically significant change in vector abundance. For example, elimination of the most productive containers could be coupled with deployment of gravid traps or egg sinks [21], [65]. Such a combined strategy may encourage females to lay eggs in traps, either for themselves (gravid traps) or for their offspring (egg sinks), as well as minimize shifts in productivity to under-utilized containers. Regardless of the specific combination of tools used, successful integrated control strategies should be based on sound understanding of *Ae. aegypti* behavior and population dynamics.

## Supporting Information

Figure S1 **Number of pupae produced per container treatment during preference-performance field experiment (mean ± SE).** Data include all 80 containers located in the 20 houses.
(TIF)

Figure S2 **Mean (± SE) wing length of males developing in four container treatments.**
(TIF)

Figure S3 **Segment plots depicting when and where individual females deposited their eggs during A) trial 1, B) trial 2, C) trial 3, and D) trial 4.** Each circle represents an oviposition container and each column represents a single day (eight containers available each day). The size of the circle corresponds to the container type, with large circles representing large unmanaged containers and small circles representing small manually filled containers. Large unmanaged containers were available only during trials 1 and 3 (pre-intervention). Containers 1–4 were located outside in the yard and containers 5–8 were inside the house. Within each trial, the same segment color and position corresponds to the same female (color wheel provided as a key). The size of the segment indicates the number of eggs laid (only those that could be genotyped). Different females were used during each trial. Females denoted with an (*) were released on day three to compensate for high female mortality during trial 2.
(TIF)

Table S1 **Air temperature, relative humidity, and water temperature at households included in field study (14 of 20 houses).** All data were recorded outdoors. Trial 1 was conducted during August 2008 and trial 2 during mid-September to mid-October 2008. * Data missing due to logger malfunction.
(DOC)

Table S2 **Dates and meteorological data for semi-field experiment examining oviposition patterns of individual females within an enclosure.** All trials took place during 2008 inside the same enclosure. ^a^ Pre-intervention scenario (two large unmanaged containers and six small manually filled containers available within enclosure). ^b^ Post-intervention scenario (eight small manually filled containers available within enclosure). ^c^ Data missing due to logger malfunction.
(DOC)
