# Supplementary figures and images for: Linking Oviposition Site Choice to Offspring Fitness in Aedes aegypti: Consequences for Targeted Larval Control of Dengue Vectors
Source: PLoS Negl Trop Dis. 2012 May 1;6(5):e1632. doi: 10.1371/journal.pntd.0001632 (PMC3341338; doi:10.1371/journal.pntd.0001632)

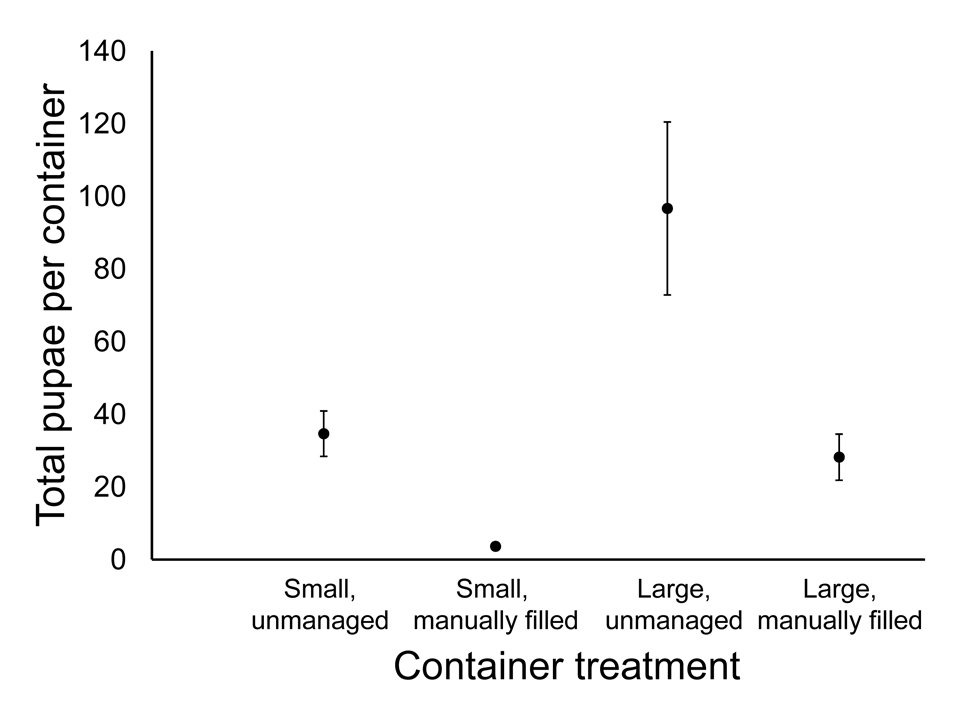

Supplement: Figure S1 — Number of pupae produced per container treatment during preference-performance field experiment (mean ± SE). Data include all 80 containers located in the 20 houses. (TIF) [file pntd.0001632.s001.tif]

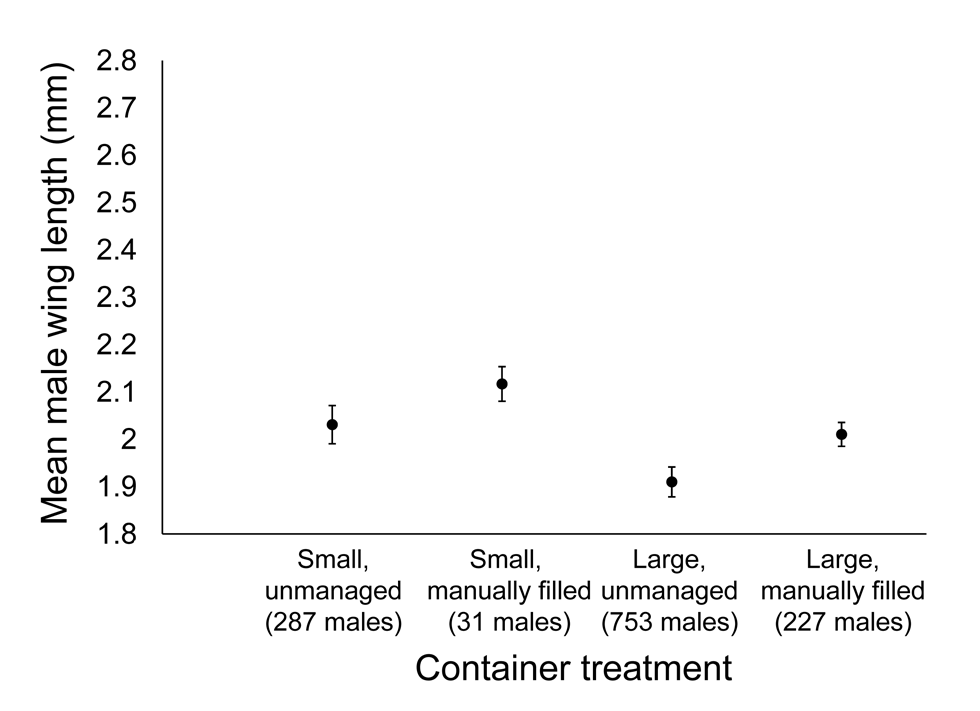

Supplement: Figure S2 — Mean (± SE) wing length of males developing in four container treatments. (TIF) [file pntd.0001632.s002.tif]

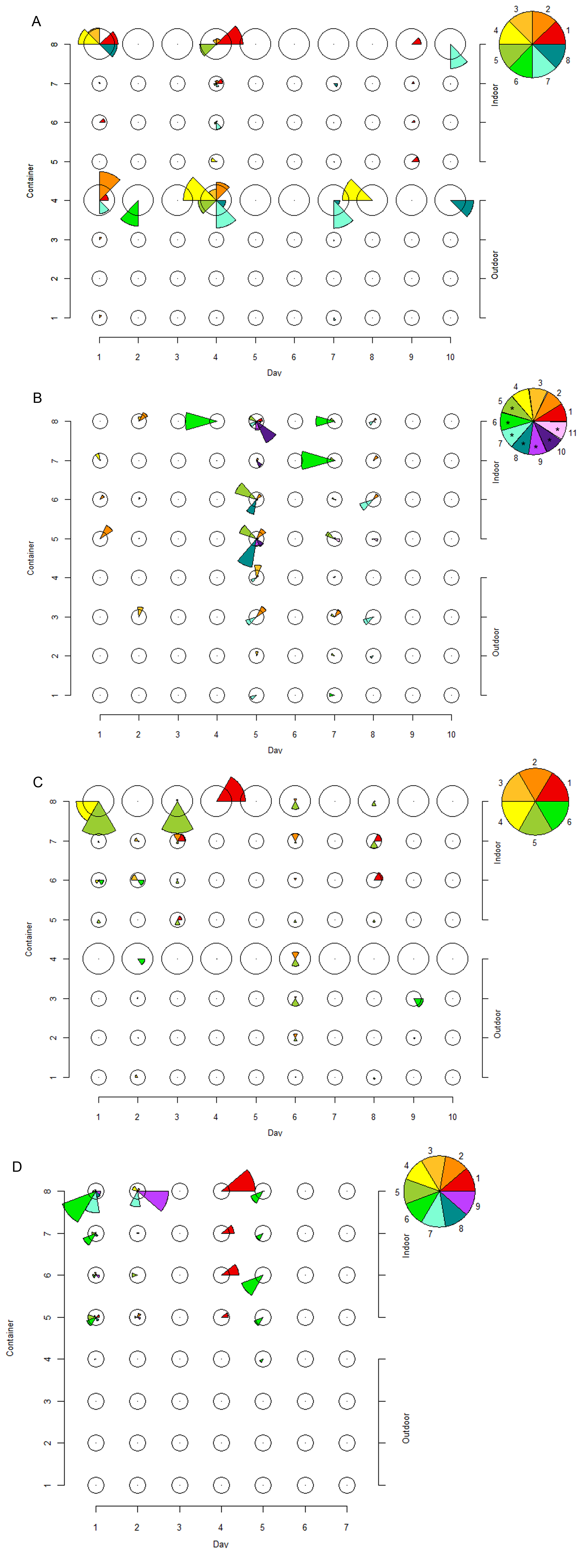

Supplement: Figure S3 — Segment plots depicting when and where individual females deposited their eggs during A) trial 1, B) trial 2, C) trial 3, and D) trial 4. Each circle represents an oviposition container and each column represents a single day (eight containers available each day). The size of the circle corresponds to the container type, with large circles representing large unmanaged containers and small circles representing small manually filled containers. Large unmanaged containers were available only during trials 1 and 3 (pre-intervention). Containers 1–4 were located outside in the yard and containers 5–8 were inside the house. Within each trial, the same segment color and position corresponds to the same female (color wheel provided as a key). The size of the segment indicates the number of eggs laid (only those that could be genotyped). Different females were used during each trial. Females denoted with an (*) were released on day three to compensate for high female mortality during trial 2. (TIF) [file pntd.0001632.s003.tif]
