# Supplementary material for: Linking Oviposition Site Choice to Offspring Fitness in Aedes aegypti: Consequences for Targeted Larval Control of Dengue Vectors
Source: PLoS Negl Trop Dis. 2012 May 1;6(5):e1632. doi: 10.1371/journal.pntd.0001632 (PMC3341338; doi:10.1371/journal.pntd.0001632)
Supplement: Table S1 — Air temperature, relative humidity, and water temperature at households included in field study (14 of 20 houses). All data were recorded outdoors. Trial 1 was conducted during August 2008 and trial 2 during mid-September to mid-October 2008. * Data missing due to logger malfunction. (DOC) [file pntd.0001632.s004.doc]

**Table S1.** Air temperature, relative humidity, and water temperature at households included in field study (14 of 20 houses). All data were recorded outdoors. Trial 1 was conducted during August 2008 and trial 2 during mid-September to mid-October 2008.

|  |  | Air temperature (°C ± SD) | | | RH (% ± SD) | Water temperature (°C ± SD) | | |
| --- | --- | --- | --- | --- | --- | --- | --- | --- |
| Household | Trial | Min | Mean | Max | Mean | Min | Mean | Max |
| 1 | 1 | 24.4 ± 0.7 | 28.1 ± 1.2 | 39.5 ± 4.7 | 77.7 ± 5.6 | 25.7 ± 0.9 | 27.7 ± 0.9 | 32.4 ± 3.0 |
| 2 | 1 | 22.6 ± 0.5 | 28.0 ± 1.3 | 40.4 ± 3.9 | 78.8 ± 6.2 | 24.9 ± 0.7 | 28.4 ± 1.1 | 34.9 ± 2.8 |
| 3 | 1 | 23.1 ± 0.6 | 28.1 ± 1.4 | 40.0 ± 4.4 | 76.3 ± 6.1 | 24.7 ± 0.9 | 28.3 ± 1.4 | 35.4 ± 3.8 |
| 4 | 1 | 23.4 ± 0.6 | 27.4 ± 1.1 | 33.5 ± 2.2 | 79.6 ± 6.2 | 24.8 ± 0.7 | 26.7 ± 0.7 | 28.9 ± 0.8 |
| 5 | 1 | 22.7 ± 0.5 | 28.0 ± 1.2 | 39.6 ± 2.8 | 77.2 ± 5.7 | 24.7 ± 0.7 | 27.8 ± 0.8 | 32.8 ± 2.2 |
| 6 | 1 | 23.1 ± 0.4 | 27.2 ± 0.9 | 33.4 ± 1.8 | 80.7 ± 4.8 | 25.0 ± 0.6 | 27.0 ± 0.7 | 29.4 ± 1.0 |
| 7 | 1 | * | * | * | * | 25.1 ± 0.9 | 28.5 ± 1.3 | 34.0 ± 3.2 |
| 8 | 2 | 23.5 ± 1.2 | 28.1 ± 2.7 | 35.7 ± 5.4 | 77.4 ± 8.2 | 24.8 ± 1.5 | 27.6 ± 2.2 | 30.8 ± 3.1 |
| 9 | 2 | 23.1 ± 1.0 | 26.6 ± 1.9 | 31.3 ± 3.1 | 81.6 ± 6.2 | 24.6 ± 1.2 | 26.3 ± 1.5 | 27.9 ± 1.7 |
| 10 | 2 | 23.6 ± 1.1 | 26.8 ± 1.8 | 31.0 ± 3.0 | 82.8 ± 6.1 | 24.9 ± 1.1 | 26.3 ± 1.2 | 27.9 ± 1.5 |
| 11 | 2 | 23.6 ± 1.1 | 27.2 ± 1.9 | 32.6 ± 3.1 | 78.8 ± 5.7 | 25.0 ± 1.3 | 26.9 ± 1.5 | 29.0 ± 1.8 |
| 12 | 2 | 24.0 ± 1.1 | 27.3 ± 1.7 | 32.0 ± 2.8 | 79.5 ± 5.3 | 25.1 ± 1.2 | 26.7 ± 1.3 | 28.2 ± 1.4 |
| 13 | 2 | 23.3 ± 1.2 | 27.2 ± 2.1 | 33.6 ± 4.0 | 79.1 ± 7.2 | * | * | * |
| 14 | 2 | * | * | * | * | 25.0 ± 1.3 | 26.4 ± 1.4 | 27.8 ± 1.6 |

* Data missing due to logger malfunction
