# Supplementary material for: Linking Oviposition Site Choice to Offspring Fitness in Aedes aegypti: Consequences for Targeted Larval Control of Dengue Vectors
Source: PLoS Negl Trop Dis. 2012 May 1;6(5):e1632. doi: 10.1371/journal.pntd.0001632 (PMC3341338; doi:10.1371/journal.pntd.0001632)
Supplement: Table S2 — Dates and meteorological data for semi-field experiment examining oviposition patterns of individual females within an enclosure. All trials took place during 2008 inside the same enclosure. a Pre-intervention scenario (two large unmanaged containers and six small manually filled containers available within enclosure). b Post-intervention scenario (eight small manually filled containers available within enclosure). c Data missing due to logger malfunction. (DOC) [file pntd.0001632.s005.doc]

**Table S2.** Dates and meteorological data for semi-field experiment examining oviposition patterns of individual females within an enclosure. All trials took place during 2008 inside the same enclosure.

|  |  | Indoors | | |  | Outdoors | | |
| --- | --- | --- | --- | --- | --- | --- | --- | --- |
|  |  |  | Air temp | Water temp | RH | Air temp | Water temp | RH |
| Trial | Dates |  | (°C ± SD) | (°C ± SD) | (% ± SD) | (°C ± SD) | (°C ± SD) | (% ± SD) |
| 1^a^ | Sept 6- Sept 1 | Min | 25.5 ± 0.5 | c |  | 24.4 ± 0.7 | 25.0 ± 0.7 |  |
|  |  | Mean | 27.3 ± 0.8 | c | 77.6 ± 2.5 | 26.4 ± 1.2 | 26.5 ± 1.0 | 83.6 ± 4.9 |
|  |  | Max | 29.3 ± 1.3 | c |  | 28.1 ± 1.5 | 27.8 ± 1.1 |  |
| 2^b^ | Sept 27- Oct 6 | Min | 26.6 ± 0.9 | 26.8 ± 0.9 |  | 26.3 ± 0.9 | 26.6 ± 0.9 |  |
|  |  | Mean | 29.3 ± 1.0 | 28.9 ± 0.8 | 72.6 ± 3.3 | 29.7 ± 1.3 | 29.5 ± 1.1 | 70.8 ± 4.8 |
|  |  | Max | 31.9 ± 1.2 | 30.7 ± 0.9 |  | 33.5 ± 2.4 | 33.3 ± 2.2 |  |
| 3^a^ | Oct 11- Oct 20 | Min | 26.2 ± 0.5 | 26.7 ± 0.7 |  | 25.0 ± 0.8 | 26.3 ± 0.9 |  |
|  |  | Mean | 27.8 ± 1.0 | 27.7 ± 0.9 | 78.1 ± 2.9 | 27.8 ± 1.4 | 27.9 ± 1.6 | 79.2 ± 4.5 |
|  |  | Max | 30.0 ± 1.5 | 28.9 ± 1.4 |  | 31.6 ± 1.8 | 30.3 ± 4.0 |  |
| 4^b^ | Oct 30- Nov 5 | Min | 25.8 ± 0.8 | 26.0 ± 0.7 |  | c | 25.3 ± 0.9 |  |
|  |  | Mean | 27.8 ± 1.0 | 27.4 ± 0.9 | 78.4 ± 2.7 | c | 27.5 ± 1.6 | c |
|  |  | Max | 30.3 ± 1.3 | 28.9 ± 1.1 |  | c | 30.4 ± 3.4 |  |

^a^ Pre-intervention scenario (two large unmanaged containers and six small manually filled containers available within enclosure)

^b^ Post-intervention scenario (eight small manually filled containers available within enclosure)

^c^ Data missing due to logger malfunction
